# EVALUATION OF SURGICAL TREATMENT OF PATIENTS WITH SHOULDER INSTABILITY

**DOI:** 10.1590/1413-785220172506166548

**Published:** 2017

**Authors:** ROBERTO YUKIO IKEMOTO, JOEL MURACHOVSKY, LUIS GUSTAVO PRATA NASCIMENTO, ROGERIO SERPONE BUENO, LUIZ HENRIQUE OLIVEIRA ALMEIDA, CLAUDIO KOJIMA

**Affiliations:** 1. Faculdade de Medicina do ABC, Departamento de Ortopedia e Traumatologia, Santo André, São Paulo, SP, Brazil.; 2. Hospital Ipiranga, Departamento de Ortopedia e Traumatologia, Ipiranga, São Paulo, SP, Brazil.

**Keywords:** Orthopedic procedures, Arthroscopy, Bankart Lesions, Shoulder joint., Procedimentos ortopédicos, Artroscopia, Lesões de Bankart, Articulação do ombro.

## Abstract

**Objective:**

To evaluate the results of arthroscopic surgery in patients with traumatic anterior shoulder dislocation.

**Methods:**

This retrospective study analyzed 76 patients with a mean age of 28 and mean postoperative follow-up period of 62 months. Evaluation consisted of physical examination, and X-rays; results were classified according to the UCLA and Rowe scales.

**Results:**

Patients showed decrease of range of motion in all planes, except elevation and lateral rotation with 90º abduction. According to the Rowe score, significant postoperative improvement was found compared with preoperative evaluations, with 89.4% of satisfactory results. According to the UCLA score, good or excellent results were observed in 97.4% of the cases. We found a 6.5% rate of recurrence.

**Conclusion:**

Arthroscopic treatment for traumatic anterior shoulder dislocation is effective, as long as indications are used. **Level of Evidence IV, Case Series.**

## RESUMO

**Objetivo:**

Avaliar os resultados da cirurgia artroscópica em pacientes com instabilidade traumática anterior do ombro.

**Métodos:**

Realizamos um estudo retrospectivo de 76 pacientes, com média etária de 28 anos e tempo médio de seguimento pós-operatório de 62 meses. A avaliação foi feita por meio de exame físico, radiográfico e classificação de resultados segundo as escalas funcionais da UCLA e Rowe.

**Resultados:**

Os pacientes apresentaram perda de amplitude de movimento em todos os planos, exceto elevação e rotação lateral em abdução de 90º. Na avaliação da escala de Rowe, observamos, em média, melhora estatisticamente significante dos resultados pós-operatórios comparadas às avaliações pré-operatórias, com 89,4% de resultados satisfatórios. Pela escala UCLA, observamos resultados satisfatórios em 97,4% dos casos. Encontramos um índice de recidiva de 6,5%.

**Conclusão:**

A cirurgia artroscópica para o tratamento da instabilidade traumática anterior do ombro é um método eficaz, desde que se respeitem as indicações. **Nível de Evidência IV, Série de Casos.**

## INTRODUCTION

Primary anterior dislocation of the shoulder after trauma is a common injury, with a frequency of 0.5% to 1.7% of the population.^1^ When it occurs in young patients, recurrence is seen in up to 90% of cases.^2^

Recent advances in arthroscopy and the growing experience of surgeons have contributed to improved results from treatment utilizing arthroscopic views to treat shoulder instability.^3^

The development of the suture anchor technique has permitted all fixations for Bankart repair to be completed using intra-articular sutures.^4^

However, according to Burkhart and De Beer,^5^ the acceptable limit for bone deficiency in the anterior-inferior glenoid bone where labral-capsular repair can be done is 25% of its diameter; this repair has a high rate of recurrence when glenoidal bone injury exceeds 25%.^5^

The following criteria are favorable for arthroscopic repair: first episode of dislocation, traumatic instability, patients over 25 years of age, presence of Bankart lesion. Adverse criteria are presence of laxity, practitioner of contact sports, patients under 25 years of age, bone injury of more than 25%, and surgical revision.^6^

Although many surgeons use the arthroscopic technique and obtain good results, this method remains controversial since recurrence rates are considered high.^7^ However, some studies have shown that the results for open and arthroscopic surgery are similar when these techniques are correctly indicated.^8^

The objective of this study is to evaluate the results of arthroscopic repair for anterior instability of the shoulder in patients with at least two years of follow-up.

## MATERIALS AND METHODS

Between February 2002 and December 2010, 101 patients underwent arthroscopic surgery to treat traumatic instability of the shoulder at our service. The project was registered with the institutional review board under protocol 158/2009, and all patients signed an informed consent form. Twenty-four individuals did not return for reevaluation and were not included in the study, so the sample consisted of a total of 76 individuals; 64 (84.2%) were male and 12 (15.8%) female, with a mean age of 28 years (17-60). The right side was affected in 46 patients (60.5%) and the left in 30 patients (39.5%). The dominant side was affected in 53.9% of cases. The mean postoperative follow-up period was 62 months (24-106). The mean number of dislocations prior to surgery was 10.3 (1-50). All patients had traumatic etiology.

At the preoperative evaluation, all patients were positive for the apprehension test, 54 patients (71.1%) were positive for the anterior drawer test, 7 (9.2%) were positive for the posterior drawer test, 31 (40.8%) were positive for sulcus sign, and 69 (90.8%) were positive for the relocation test.

To quantify bone loss, we took plain X-rays and computed tomography scans of the shoulder prior to surgery. We used bilateral Bernageau views in the X-ray^9^ to measure the antero-posterior diameter of the glenoidal cavity. The tomographic slices were performed in the axial plane, and in both methods the bilateral values were compared. Arthroscopic surgery was indicated when bone erosion was less than 25%. The inclusion criteria were traumatic anterior instability subjected to Bankart repair with the use of anchors, and follow-up of at least two years. Patients with uncontrolled seizures were excluded.

**Figure 1:**
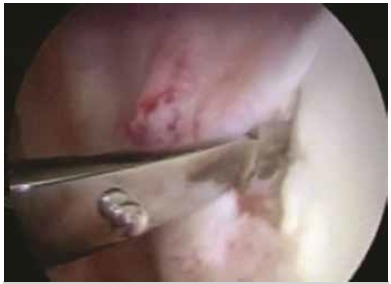
Detachment of the labrum.

**Figure 2:**
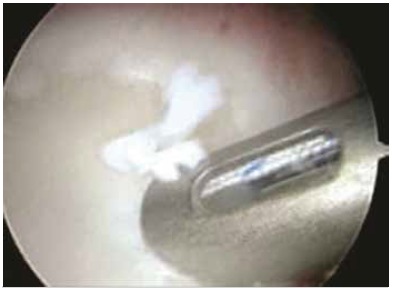
Introduction of bioabsorbable anchor.

**Figure 3:**
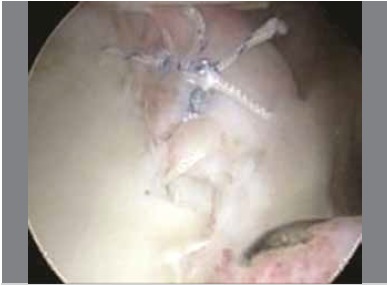
Labrum repair complete.

The surgeries were performed with the patient in lateral decubitus, under general anesthesia combined with a brachial plexus block; the operated limb was placed in traction. Posterior, antero-superior, and antero-inferior arthroscopic portals were opened, and Bankart lesions were seen in 73 patients (96.1%), SLAP type 1 injury in 4 patients (5.3%), SLAP type 2 in 3 patients (3.9%), SLAP type 4 in 1 patient (1.3%), and ALPSA lesion in 3 cases (3.9%). (Table 1 and Figure 1) Of the total number of patients, 62 (81.6%) had no injury to the glenoid, and 14 (18.4%) exhibited damage to the glenoid (Table 2); the average lesion size was 14.43%(10%-20%). To repair the injury, we used two anchors in 6 patients (7.9%), three anchors in 58 patients (76.3%), and four anchors in 12 patients (15.8%); in 25 patients (32.9%) we used bioabsorbable anchors, and in 51 patients (67.1%) we used metallic anchors. (Figures 2 and 3) After the procedure, the patients kept the operated limb immobilized in a sling and performed exercises for elbow flexion-extension, swinging, and passive/active external rotation to neutral. After four weeks, the immobilization was discontinued and the patients began exercises to gain mobility, and muscle strengthening was started in the third month. (Tables 1 and 2)

**Table 1:** Intra-operative findings.

Finding	n	% (in 76 cases)
Bankart	73	96.1
Slap 1	4	5.3
Slap 2	3	3.9
Slap 4	1	1.3
ALPSA	3	3.9

**Table 2:** Bone erosion.

Injury	n	%
Yes	14	18.4
No	62	81.6
Total	76	100.0

Clinical evaluation in the postoperative period consisted of: measuring the entire range of motion of the shoulders to compare whether there was restricted mobility, the anterior apprehension test, X-ray evaluation in the corrected AP and axilla positions to diagnose signs of arthrosis (degrees were determined according to the classification by Samilson and Prietto^10^), and the functional scales of by Rowe^11^ and UCLA,^12^ comparing pre- and postoperative values.

Statistical analysis of the results was performed using SPSS (Statistical Package for Social Sciences) version 15.0 software, adopting a 5% significance level. All variables were analyzed descriptively. For quantitative variables, this was done by observing minimum and maximum values and calculating the means, standard deviations, and medians. For the qualitative variables, absolute and relative frequencies (%) were calculated. Student’s t-test was used to compare the means of the two groups, and when the assumption of normality was rejected, we used the non-parametric Mann-Whitney test. To test homogeneity between the proportions we used the chi-squared test or Fisher’s exact test (when there were expected frequencies lower than 5). To compare pre- and post-surgery, we used the paired Student’s t-test.

## RESULTS

Assessment of the range of motion between the operated and non-operated shoulders showed a statistically significant decrease in lateral rotation (69° vs. 63.3º) (p=0.002), medial rotation (T5 vs. T6) (p<0.001), and medial rotation in 90º abduction (78.6º vs. 77.3º) (p=0.004). For elevation and lateral rotation in 90° abduction, although there was a decrease in the postoperative period this was not statistically significant (p=0.219).

According to the Rowe scale, a statistically significant improvement was seen between the pre- and postoperative periods: a mean of 39.9 in the preoperative and 91.5 in the postoperative period (p<0.001; 8 cases were poor (10.6%), 2 cases were good (2.6%), and 66 cases (86.8%) were excellent. There was also a statistically improvement between the pre- and postoperative periods according to the UCLA scale. The average pre-surgery score was 27.8 and postoperative score was 33.4 (p<0.001); 2 cases were regular (2.6%), 7 (9.2%) were good, and 67 (88.2%) were excellent.

**Figure 4:**
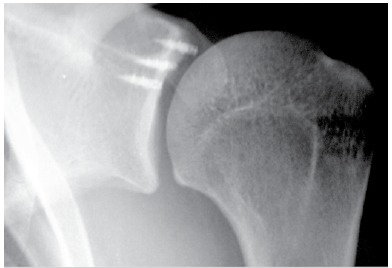
Metallic intra-articular anchor.

We found 11 cases (14.5%) with intra-operatory complications: 1 broken bioabsorbable anchor (1.3%), 7 anchor losses (9.2%), 1 inability to repair the labrum (1.3%), 1 breach of the impactor (1.3%), and 1 protruding anchor (1.3%). Postoperative complications occurred in 22 patients (28.9%), 4 cases of recurrence (5.3%), 12 cases of arthrosis (15.7%), 5 cases of anchor extrusion (6.5%), (Figure 4) 2 cases of adhesive capsulitis (2.6%), and 1 superficial infection (1.3%).

As for the physical examination, the four patients who developed recurrent dislocation in the postoperative period were positive for the apprehension test.

There was no association between recurrence and intra-operative complications, according to Fisher’s exact test (p = 1.000). No association was seen between recurrence and anchor type (Fisher’s exact test, p=1.000). The number of episodes of dislocations had no statistical relationship with postoperative recurrence (Mann-Whitney non-parametric test, p=0.559). There was also no association between the number of episodes and postoperative arthrosis (Mann-Whitney non-parametric test, p=0.720). No relationship was seen between the number of anchors and recurrence (Fisher’s exact test, p=0.381). There was also no relationship between recurrence and bone erosion (Fisher’s exact test, p=0.172).

Presence of ALPSA-type injury was not a determining factor for recurrence (Fisher’s exact test, p=1.000).

Furthermore, no statistical differences were seen between intra-operative and late complications with regard to anchor type (Table 1) (Fisher’s exact test, p=1.000 and p=0.123, respectively). On the other hand, we observed that the groups differed when we compared cases with pain and type of anchor used. In the 18 patients (23.7%) who presented pain, 2 cases (8%) received bioabsorbable anchors and 16 (31.4%) received metal anchors (descriptive level of probability of the chi-square test, p=0.024).

No statistically significant relationship was seen between the number of anchors and arthrosis (Fisher’s exact test, p=0.009). No association was seen when arthrosis was compared with intra- operative complications (Fisher’s exact test, p=0.668) and late complications (Fisher’s exact test, p=0.080). No association was observed between pain and intra-operative complications (Fisher’s exact test, p=0.716), but an association was seen between pain and late complications (Fisher’s exact test, p=0.005). (Table 3)

**Table 3:** Relation between complication by type of anchor.

Complication	Type	Bioabsorbable	Metallic
Intra-operative	Breakage of anchor	1	-
	Loss of anchor	2	5
	Impossible to repair the labrum	-	1
	Impactor breakage	-	1
	Protruding anchor	-	1
Late	Recurrence	1	3
	Arthrosis	1	11
	Protruding anchor	-	5
	Adhesive capsulitis	-	2
	Subluxation	-	1
	Infection	1	-

As for X-ray assessment in the postoperative follow-up, 12 cases (15.8%) presented evidence of arthrosis. According to the classification by Samilson and Prietto, 9 were classified as grade I (11.8%) and 3 as grade II (3.9%).

## DISCUSSION

Open stabilization has a higher success rate, with a lower incidence of recurrence and less potential for complications when compared with arthroscopy.^3^ However, if patients are carefully selected, the results may be equivalent.^13^ In our study, all patients had bone erosion below 25%, which is the limit for arthroscopic repair according to the literature.^10^

Ferreira Neto et al.^14^ obtained 10% recurrence in 159 patients, and Carreira et al.^15^ had 10% recurrence of instability in 85 patients; Marquardt et al.^16^ obtained 7.5% recurrence in 54 patients. Although the literature discusses greater chances of recurrence, we observed 4 cases (5.3%), and these were the same patients who continued to have a positive apprehension test.

In this study, the use of metal anchors (67.1%) was related to the presence of residual pain. This fact agrees with the literature; Jeong and Shin^17^ assessed 43 patients, noting 33% of cases of residual pain associated with the use of metal anchors. In our study, we found 6 cases in which the anchor required subsequent removal.

Our study found a statistically significant reduction in lateral rotation, medial rotation in neutral, and medial rotation in 90° abduction. In the literature, no study observed a loss of range of motion.^17^ However, the reduction of amplitudes did not result in compromised clinical and functional outcome. As for X-ray assessment, 15.8% of cases showed signs of arthrosis, justified by the osteochondral lesion associated with instability.^18^

In the Rowe score assessment, we observed a statistically significant improvement when comparing the pre- and postoperative means, with 89.4% attaining good results. In a study of 53 patients, Gartsman et al.^19^ obtained 91.9 points for the Rowe score. Boileau et al.^20^ presented the results of 91 patients who underwent surgery and were and evaluated according to the Rowe criteria, and obtained an average score of 77.8 points. Balg and Boileau^13^ analyzed the results of 131 patients who were evaluated according to the Rowe criteria, and found an average score of 81.5 points.

In our study, it was not possible to correlate fewer anchors and recurrence, because few cases used only two anchors (7.9%); similarly, the presence of ALPSA-type lesions (3.9%) was not associated with recurrence, but according to the literature fewer anchors and ALPSA-type injury are related to recurrence.^20^

Although our results regarding recurrence have demonstrated compatibility with the values found in the literature, it is possible that, in an attempt to prevent recurrence in these severe cases by repairs to the labrum and plication, some range of motion was lost in these patients.

## CONCLUSION

Arthroscopic surgery is an effective method for treating traumatic anterior instability in the shoulder. After a follow-up of at least two years, we observed a recurrence rate of 5.3%, which is established in the literature.

In this study, the use of metal anchors is associated with greater pain in the postoperative period.

## References

[1] Hovelius L (1982). Incidence of shoulder dislocation in Sweden. Clin Orthop Relat Res.

[2] Hovelius L (1978). Shoulder dislocation in Swedish ice hockey players. Am J Sports Med.

[3] Godinho GG, Souza JMG, Freitas JMA, Santos FML, Vieira AW, João FM (1997). Tratamento da instabilidade anterior do ombro. Rev Bras Ortop.

[4] Wolf EM (1993). Arthroscopic capsulolabral repair using suture anchors. Orthop Clin North Am.

[5] Burkhart SS, De Beer JF (2000). Traumatic glenohumeral bone defects and their relationship to failure of arthroscopic Bankart repairs significance of the inverted pear glenoid and the humeral engaging Hill-Sachs lesion. Arthroscopy.

[6] Calvo E, Granizo JJ, Fernández-Yruegas D (2005). Criteria for arthroscopic treatment of anterior instability of the shoulder a prospective study. J Bone Joint Surg Br.

[7] Lenters TR, Franta AK, Wolf FM, Leopold SS, Matsen 3rd FA (2007). Arthroscopic compared with open repairs for recurrent anterior shoulder instability A systematic review and meta-analysis of the literature. J Bone Joint Surg Am.

[8] Hobby J, Griffin D, Dunbar M, Boileau P (2007). Is arthroscopic surgery for stabilization of chronic shoulder instability as effective as open surgery A systematic review and meta-analysis of 62 studies including 3044 arthroscopic operations. J Bone Joint Surg Br.

[9] Edwards TB, Boulahia A, Walch G (2003). Radiographic analysis of bone defects in chronic anterior shoulder instability. Arthroscopy.

[10] Samilson RL, Prieto V (1983). Dislocation arthropathy of the shoulder. J Bone Joint Surg Am.

[11] Rowe CR, Patel D, Southmayd WW (1978). The Bankart procedure a long-term end result study. J Bone Joint Surg Am.

[12] Amstutz HC, Sew Hoy AL, Clarke IC (1981). UCLA anatomic total shoulder arthroplasty. Clin Orthop Relat Res.

[13] Balg F, Boileau P (2007). The instability severity index score A simple pre-operative score to select patients for arthroscopic or open shoulder stabilisation. J Bone Joint Surg Br.

[14] Ferreira AA, Camanho GL, Felix AM, Benegas E, Bitar AC, Ramadan LB (2011). Tratamento artrosco´pico da instabilidade anterior do ombro estudo retrospectivo de 159 casos. Acta Ortop Bras.

[15] Carreira DS, Mazzocca AD, Oryhon J, Brown FM, Hayden JK, Romeo AA (2006). A prospective outcome evaluation of arthroscopic Bankart repairs minimum 2-year follow-up. Am J Sports Med.

[16] Marquardt B, Witt KA, Liem D, Steinbeck J, Pötzl W (2006). Arthroscopic Bankart repair in traumatic anterior shoulder instability using a suture anchor technique. Arthroscopy.

[17] Jeong JH, Shin SJ (2009). Arthroscopic removal of proud metallic suture anchors after Bankart repair. Arch Orthop Trauma Surg.

[18] Brophy RH, Marx RG (2005). Osteoarthritis following shoulder instability. Clin Sports Med.

[19] Gartsman GM, Roddey TS, Hammerman SM (2000). Arthroscopic treatment of anterior-inferior glenohumeral instability Two to five-year follow-up. J Bone Joint Surg Am.

[20] Boileau P, Villalba M, Héry JY, Balg F, Ahrens P, Neyton L (2006). Risk factors for recurrence of shoulder instability after arthroscopic Bankart repair. J Bone Joint Surg Am.

